# *Mycobacterium abscessus* Infections in Cystic Fibrosis Individuals: A Review on Therapeutic Options

**DOI:** 10.3390/ijms24054635

**Published:** 2023-02-27

**Authors:** Deborah Recchia, Giovanni Stelitano, Alessandro Stamilla, Damaris L. Gutierrez, Giulia Degiacomi, Laurent R. Chiarelli, Maria Rosalia Pasca

**Affiliations:** Department of Biology and Biotechnology “L. Spallanzani”, University of Pavia, 27100 Pavia, Italy

**Keywords:** *Mycobacterium abscessus*, alternative strategies, nitric oxide, phage therapy, antivirulence compounds, antimicrobial peptides, phytochemicals, drug delivery

## Abstract

*Mycobacterium abscessus* is an opportunistic pathogen that mainly colonizes and infects cystic fibrosis patients’ lungs. *M. abscessus* is naturally resistant to many antibiotics such as rifamycin, tetracyclines and β-lactams. The current therapeutic regimens are not very effective and are mostly based on repurposed drugs used against *Mycobacterium tuberculosis* infections. Thus, new approaches and novel strategies are urgently needed. This review aims to provide an overview of the latest ongoing findings to fight *M. abscessus* infections by analyzing emerging and alternative treatments, novel drug delivery strategies, and innovative molecules.

## 1. Introduction

Cystic fibrosis (CF) is one of the most common serious genetic conditions. Formerly called mucoviscidosis, CF is an autosomal recessive genetic disease caused by mutations in the CFTR (Cystic Fibrosis Transmembrane Regulator) gene. CF is a multi-organ disease affecting organs such as the pancreas, liver, reproductive tract, and lungs [1]. The CFTR gene, found on chromosome 7, encodes a ubiquitous transmembrane protein that regulates the passage of electrolytes (chlorine in particular) and water from the inside to the outside of epithelial cells. The loss of function of CFTR in the lungs causes several collateral effects, such as the presence of sticky mucus, which leads to insufficient mucociliary clearance and dysfunction in the phagocytosis process [2,3]. This condition leads to chronic bacterial infections and to colonization by opportunistic pathogens such as *Pseudomonas aeruginosa* and Nontuberculous mycobacteria (NTM) such as *Mycobacterium abscessus* (*Mab*) [4]. Furthermore, the mucus tends to stagnate inside the bronchi and lungs, generating inflammation; these conditions, worsening over time, lead to respiratory failure. 

In addition to the respiratory symptoms, CF is often associated with pancreatic insufficiency, which results in defective digestion, diarrhoea, malabsorption, growth retardation in children, and poor nutritional status in adults [5]. Other manifestations may involve the intestines, liver, nasal cavities, and vas deferens. The sweat glands, which produce very salty sweat, are normally compromised, making sweat testing, consisting of the measurement of chloride concentration in sweat, critical to diagnosis [6].

The life span of CF individuals mainly depends on the evolution of the lung disease [5], which is determined by a combination of genetic and environmental factors. The main genetic factors are the type of CFTR gene mutations; so far, more than 2000 of these gene mutations have been reported [7,8]. Additionally, the presence of variants of other genes called modifiers, as suggested by various studies [9,10], can positively or negatively interact with the CFTR gene. Among the environmental factors, the type of treatments, the level of adherence, and the lifestyle adopted are also considered.

Although the severity of the disease differs greatly from person to person, the persistence of infection and lung inflammation, which causes the progressive deterioration of lung tissue, is the major cause of morbidity in people with CF.

There are different forms of CF, more or less serious, which can also be identified in a more adult stage of life, comprising about 10% of total cases. In fact, CFTR genetic variants can generate a great clinical heterogeneity with different manifestations of the disease, especially in the lungs, which consequently results in different treatments and cures throughout the person’s life. CF therapy has been considerably improved in recent years. In fact, alongside symptom therapy, personalized therapies are now starting to treat the basic defect in some CFTR mutations [7]; it is hoped that, within a few years, all genetic variants could become curable.

Thanks to scientific research, new therapeutic approaches have indeed emerged that can correct the defects underlying CF. These drugs, which are called CFTR protein modulators, are able to restore CFTR function to a level of about 40–50% of normal [11]. Modulators help improve the function of the CFTR chloride and bicarbonate channel on the apical surface of epithelial cells throughout the body, thereby decreasing chloride in sweat, and improving lung function, growth, and other parameters [12]. So far, CFTR modulators have been developed to target the most common CFTR mutations. Currently, four modulator drugs are available on the market and effective in people with specific CFTR mutations: Kalydeco (whose active metabolite is called ivacaftor), Orkambi (lumacaftor/ivacaftor), Symkevi (tezacaftor/ivacaftor) and Kaftrio (elexacaftor/tezacaftor/ivacaftor) [13]. Ivacaftor was the first CFTR modulator accepted for use in people with CF targeting gating mutations, that is, mutations in which the chloride channel is closed, thus preventing chloride from leaving the cell [12]. A number of clinical trials are assessing the safety and efficacy of novel modulators targeting other CFTR mutations [7]. These innovative pharmaceutical therapies are transforming the lives of many CF patients, with improvements at the clinical level and in overall health status.

However, opportunistic infections remain the main issue for CF individuals, with those from NTM increasing alarmingly [14]. Indeed, although the most common pathogens in people with CF are *Staphylococcus aureus* and *Pseudomonas aeruginosa*, there are more than 30 species of NTM known to cause infections in humans [14,15], some of which show an increasing incidence in CF patients—leading to an increase in morbidity and mortality [16]. Immunocompromised patients, an improved life expectancy and also various factors, such as the use of inhaled corticosteroids, can contribute to such increases [17].

Among NTM, *Mycobacterium abscessus* complex (MABSC) encompasses three subspecies: *M. abscessus* subsp. *abscessus* (*Mab*), *M. abscessus* subsp. *bolletii* and *M. abscessus* subsp. *massiliense* (*M. massiliense*) [18], even though this taxonomic classification is still under discussion. Unlike other rapid-growing mycobacteria, *Mab* has adapted to survive intracellularly in human macrophages [15] and is among the main causes of morbidity and mortality in people with CF, in addition to the more common *P. aeruginosa* and *M. avium*. In these individuals, *Mab*’s natural antibiotic resistance and ability to form biofilm led to severe pulmonary infections, which were difficult to treat [19]. Misdiagnosis with *Mycobacterium tuberculosis* (*Mtb*) or other NTM occurred due to the lack of standardized criteria [20]. Moreover, it could be difficult to differentiate between transient colonization and pulmonary disease, even in the presence of a positive sputum culture. In this context, to have a more accurate diagnosis, the American Thoracic Society and the Infectious Disease Society of America published a guideline in 2007 which specified that a diagnosis of NTM pulmonary disease requires clinical symptoms, radiological and microbiological evidence, and exclusion of other diseases like tuberculosis [21,22].

The therapy for pulmonary MABSC infections remains extremely difficult, being resistant to aminoglycosides, rifamycins, tetracyclines, and β-lactams [23,24]. As a result, many treatment strategies for *Mab* infections use prolonged antimicrobial therapy, ranging from months to years, with significant side effects such as potential risks of antibiotic toxicity and a high number of failures [25]. Therapy, therefore, often must be changed or discontinued [24]. Furthermore, the current treatment recommendations for MABSC may hinge on the subspecies. For example, it was suggested that the *erm(41)* gene encoding an inducible methylase is related to a different macrolide sensitivity among the MABSC. In fact, it was shown that an isolate of *M. massiliense*, sensitive to macrolides, possesses an incomplete *erm(41)* gene. It is still not clear if reversion mutations can cause an acquired resistance in this subspecies. On the other hand, *Mab* and *M. abscessus subsp. bolletii* are usually resistant due to the presence of a functioning *erm(41)* gene [26], possibly explaining the inefficacy of macrolides for their treatment.

Therefore, it is necessary to continue to find alternatives to curb this problem. In this context, drugs already used against *Mtb* could be useful. For instance, a study conducted by Banaschewski B. et al. [27] developed a liquid suspension of clofazimine for the treatment of NTM lung disease in mouse models. This clofazimine inhalation suspension showed potent antimycobacterial activity and was well tolerated in various naïve mouse models. Another study aimed to investigate the impact of efflux pump inhibitor (EPI) activity on bedaquiline in *Mab* [28]. This study demonstrated that bedaquiline Minimal Inhibitory Concentrations (MICs) in clinical isolates of *Mab* were affected by the presence of verapamil and reserpine, suggesting the role of efflux pump (EP) activity in bedaquiline efficacy. These results are in agreement with another recently-published research work in which verapamil enhanced bedaquiline activity against *Mab* with a 4- and 8-fold reduction in bedaquiline MIC [29]. Furthermore, it was shown that bedaquiline could be used as an alternative in multidrug treatment regimens for severe or relapsing disease, potentially including patients with underlying CF [30].

To date, there is not a consolidated standard drug regimen to treat MABSC infections, although the US Cystic Fibrosis Foundation and the European Cystic Fibrosis Society published consensus recommendations for the management of NTM in patients with CF [31]. The official ATS/ERS/ESCMID/IDSA clinical practice directions [32] recommend a multidrug regimen of three or more active drugs to treat NTM, for example, combinations of tigecycline, imipenem, cefoxitin and amikacin. Importantly, a treatment including a macrolide can be given to patients infected by a macrolide-resistant strain if these compounds are used for immunomodulatory reasons. *Mab* drug therapy takes up to two years, with an average rate of treatment success of 45.6%. In addition, many guidelines recommend continuing the treatment for one year after negative sputum culture and positive sputum culture after six months of therapy is considered a treatment failure [33,34,35].

For all these reasons, the identification of new therapeutic strategies that can support or refine the scarce antibiotic options available today is an imperative issue to be addressed. The lack of effective therapeutic solutions for MABSC infections worsens the life quality and expectancy of CF individuals, representing both a diagnostic and a treatment dilemma. 

Intracellular pathogens evolved to infect, colonise, and duplicate within host cells, managing to hide from the host immune system [36,37]. Current antimicrobial drugs were developed to face extracellular pathogens which spend most of their life cycle outside the host cells. Thus, intracellular bacteria are much more difficult to treat, and the usual strategies developed for the fight against bacterial infection should be rethought.

This lack of appropriate classical therapies has different causes, including antimicrobial resistance (AMR) which could have several mechanisms, such as the misuse of antimicrobials in both the healthcare and veterinary sectors. Another cause of AMR is the poor pharmacokinetics of many classical drugs, due to difficulty in reaching the site of action; as a result, subcellular niches used by infecting bacteria are not reached by most current drugs. Finally, AMR can also be induced by the need for long periods of treatment to reach the effective circulating dose, with an inevitable increase in side effects [38]. Inevitably, many first-line antibiotics are now ineffective less than a century after their introduction [19,39].

To fight the antimicrobial-resistant and emerging pathogen *Mab*, it is important to look for alternative strategies or to repurpose existing pharmaceuticals [40]. In this review, we will focus our attention on recently-developed alternative therapeutical strategies like nitric oxide treatment, phage therapy, anti-virulence drugs, and peptides.

## 2. Nitric Oxide Therapies against *M. abscessus*

Nitric oxide (NO), a highly reactive vaso- and broncho-dilator molecule is part of the natural defence mechanism of the immune system [41,42], i.e., a lipophilic free radical that plays an essential role in host protection mechanisms against infection at various sites. Moreover, it was shown that NO represents an encouraging antimicrobial approach, especially in lung infections. 

Endogenous NO is formed by nitric oxide synthases (NOSs) using L-arginine as a precursor [43]; it is particularly involved in the control of infectious diseases, cancers, and autoimmune diseases [44]. Among host immune cells, macrophages have a similar mechanism of action as phagocytes; they engulf and destroy foreign cells. Macrophages are known to play a major role in the progressive evolution of tuberculosis disease; several studies demonstrated that they are also involved in *Mab* invasion response [45]. During infection, NO production is intensified in alveolar macrophages because of bacterial products or inflammatory stimuli [46].

It is noteworthy that CF individuals are characterised in the lungs by a low NO production [41]. As a result, a low NO concentration is measured in the air exhaled by CF persons. It could depend on the presence of the mucus layer that prevents the NO from reaching the bronchial lumen and thus from being exhaled. Another hypothesis could be that there is a shortage of substrates, like L-Arg, essentials for NO production. So, lower NO production could contribute to altered defence mechanisms and, thus, to lung damage [41]. In this context, some studies [47,48,49] aimed to investigate whether NO deficiency in CF people is due to a lack of substrates, such as arginine, as a precursor for NO production. 

A recent clinical trial [50] evaluated whether intermittent exposure to high concentrations of inhaled NO is safe and effective in CF people with chronic lung *Mab* infection. In this pilot study [50], inhaled NO was administered for 30 min, 5 times a day for the first 2 weeks, followed by 3 times a day for another week. The inhaled gas was safe, and no significant adverse events were reported. In addition, slight improvements in patients’ lung function and exercise tolerance were found. Lastly, a reduction in the amount of *Mab* cells in sputum was shown, although the bacterium was not completely eliminated [50]. However, the main limitation of this study was the low number of patients enrolled and its brevity. Therefore, the results should be interpreted with extreme caution. Researchers are planning a new clinical trial with a larger number of individuals, which will examine the effect of NO inhalation in a time interval longer than 3 weeks, to hopefully achieve the eradication of the bacterium in patients.

Other preclinical studies have observed a broad-spectrum antibacterial function after the administration of gaseous NO at a high dose (≥160 p.p.m.) [51] and that both healthy people and individuals with CF can tolerate intermittent inhalation treatment of NO at higher doses (160 p.p.m.) well and safely [52,53]. In CF individuals with pulmonary disease due to NTM, decreases in lung *Mab* burden and improvement in airway function have been also detected following intermittent administration of NO at 160 p.p.m. [50,54].

Therefore, being as this preliminary evidence very promising, it is advisable to continue to study the use of NO as an alternative strategy to treat *Mab* infection in CF patients.

## 3. Phage Therapies: Background, and Their Application in CF People with *Mab* Infection

Phage therapy (PT) represents an old idea which has acquired renewed importance thanks to the successful recent case reports, where antibiotic-resistant lung infections in CF people were treated [55].

PT refers to the use of phages to fight bacterial infections. The idea of using phages as a weapon against bacteria was advanced shortly after their discovery, almost a century ago, by Frederick Twort and Félix d’Herelle. Interestingly, the latter was able to eradicate four cases of paediatric dysentery in 1919 in Paris using the administration of a phage preparation [56,57]. In the following decades, other attempts with PT were performed, providing results not always consistent and much debated. Indeed, in the 1940s, upon the introduction of the first antibiotic, PT was abandoned [58], especially in Western countries [57].

Therapeutic bacteriophages are pathogen-specific and safe for human tissues [59]. In addition, bacteriophages are the most abundant microorganisms and are distributed in soil, water, and air in different ecosystems and surfaces both inside and outside human and animal bodies. 

In recent years, there has been a renewed interest in the Western world in phage therapy as an alternative or addition to antibiotic therapy.

Currently, there have been few reported cases of phage therapy in humans, e.g., a clinical trial done in London reached phase II for the treatment of chronic otitis from *P. aeruginosa*; a case of severe sepsis caused by *Acinetobacter baumannii* in the USA; and a case of septicaemia from *P. aeruginosa* in Belgium [60,61,62].

Of particular interest is a study carried out in Tbilisi (Georgia), in which CF people with pulmonary infections were treated by nebulization with phage preparations, combined with conventional antibiotics, anti-mucus drugs, and vitamins. This showed, in all cases, an improvement in the conditions, without significant side effects [63].

PT was used for the first time against mycobacteria in 2019 by Dedrick et al. [64], who successfully reported the treatment of a 15-year-old CF patient. The patient showed several co-morbidities and was chronically infected with *P. aeruginosa* and *M. massiliense*; so, she was treated with an anti-NTM therapy for 8 years before lung transplantation. Nevertheless, a disseminated mycobacterial infection occurred a few months after the transplant. A cocktail of three ad hoc bacteriophages was isolated and engineered, and then administered intravenously. The bacteriophage treatment was associated with objective clinical improvement, and without significant adverse effects, suggesting that PT may be a viable alternative and a practical solution for MABSC infection [63]. 

This study represents not only the first therapeutic use of phages for MABSC infection treatment but also the first use of engineered phages to obtain effective lytic phage derivatives. However, the use of phages in clinical therapy presents some potential challenges. One possible drawback is the extreme specificity of phages in targeting bacteria; thus, bacterial susceptibility is needed prior to choosing the suitable phage(s) [56]. 

Furthermore, a variation in MABSC phage susceptibilities was found [63], thus the PT of similar infections could be different; for this reason, a deeper understanding of phage infection will be needed. 

The most important issue to solve is the possible resistance to phages induced by the treatment. Recently, Dedrick et al. [65] treated 20 patients with PT intravenously and by aerosolization for compassionate use. Favourable outcomes were observed in 11 patients. Unfortunately, 8 patients developed neutralizing antibodies after the initiation of phage delivery intravenously. Preclinical and clinical evaluation studies are still needed before phage-based therapies administration, particularly regarding the possible production of neutralising antibodies. PT appears to be an alternative approach against *Mab* infections, also in combination with antibiotics.

## 4. Antivirulence Therapies

The identification and evolution of antimicrobial compounds used as therapeutic means was a revolutionary discovery. However, while humans have long benefited from antimicrobials, mechanisms of resistance to classical antimicrobials have emerged and spread among bacteria [66]. 

Hence, antivirulence therapy (AVT) consists of the use of drugs targeting pathways important for pathogenesis but not essential for microbial growth [38]; it is considered an alternative strategy against AMR in contrast to current cidal or static antimicrobials that target functions essential for microbial growth. Indeed, pathogens can adopt an arsenal of virulence factors (VFs) to establish themselves in their infectious niche; consequently, it has been shown that AVT targeting VFs, or their production/regulation, could be a potential substitute or adjuvant to conventional antimicrobial drugs [66].

Selective pressure is not a consequence of the AVT approach, as it aims to prevent attacks on the host rather than eliminate pathogens. This should not allow drug resistance to develop [67].

The horizontal gene transfer and the consequent transmission of resistance genes should not be an issue in AVT, in fact, most VFs are present in a few closely related species. Another strength of AVT is that this approach should keep the host microbiota healthy, lacking the undesirable side effects typical of antibiotic therapy [67]. 

Some limitations in the use of AVT are, however, to be expected. For instance, the simultaneous presence of several redundant VFs in some species could only be addressed by the combined use of different compounds. In addition, the timing of treatment administration must coincide with the regulation and the subsequent production of the target factor during the infection process. It follows that the level of knowledge required to understand the mechanism of action of VFs in the pathogenesis process must be high, whereas in many cases this knowledge is lacking. The site of infection may also play a role in the expression of VFs, varying it accordingly [67]. Moreover, rapid diagnostics are needed for the implementation of AVT [66]. The use of antivirulence compounds in combination with antibiotics can be an effective strategy, but in this case, pharmacokinetic and pharmacodynamic parameters must be considered, due to possible drug interactions. For all these reasons, the mechanism of action of each VF has to be studied and analysed to achieve a successful AVT [67]. Hence, despite the potential of AVT, most of the compounds that have been developed are still in preclinical development.

Despite these limitations, currently, AVT compounds against *Mab* have not yet been reported, mainly since the current knowledge of *Mab* virulence mechanisms is still lacking. However, it is worth noting that some virulence mechanisms are common to all mycobacteria, including *Mtb*, for which research is at a much more advanced stage [68]. Indeed, *Mtb* has evolved by employing multiple strategies to avoid the host’s efficient immune response. For example, anti-apoptosis genes have been identified in the *Mtb* genome, giving the possibility of studying this host-pathogen interaction in detail, although the apoptosis mechanism in humans is not fully defined [69]. Like *Mtb*, *Mab* can proliferate intracellularly; therefore, it has the ability to avoid the host’s defences [68]. These common pathways can represent the starting point for the development of specific antivirulence compounds against *Mab*. 

To understand bacteria from a pathophysiological perspective, it may be important to obtain information on both the complete sets of causative genetic variants and the complex gene–gene (or “epistatic”) interactions [67]. For example, to understand the system-level *Mab* pathobiology, Boeck et al. employed genome-wide association studies (GWAS), informed by computational structural modelling of the entire proteome, for a broad spectrum of in vivo, in vitro, and clinical traits, giving confirmation of known genetic associations for antibiotic resistance and uncovering many unknown genotype–phenotype associations, many of which have been experimentally validated [70]. The authors discovered three clusters characterized by different virulence traits and associated with a different clinical outcome, by analyzing 331 *Mab* clinical isolates. Then, they used a CRISPR knockdown of linked genes in both in vitro and in vivo infection assays, with the final aim of discovering several mycobacterial VFs. In particular, two genes, encoding a putative secretion system protein (MAB_0471) and a non-ribosomal peptide synthetase (MAB_3317c), regulate *Mab* virulence; so, they could be possibly potential AVT targets.

## 5. Antimicrobial Peptides

Among the most recent discoveries, antimicrobial peptides (AMPs) are becoming an increasingly promising weapon against bacterial infections. AMPs are short polypeptides, generally smaller than 100 amino acids, which are rich in lysin, arginine, and hydrophobic residues, and show antibacterial activity and immunomodulatory properties [71,72].

Recent studies demonstrated the efficacy of some AMPs against *Mab* when used either alone or in combination with antibiotics showing a synergic effect.

**Figure 1:**
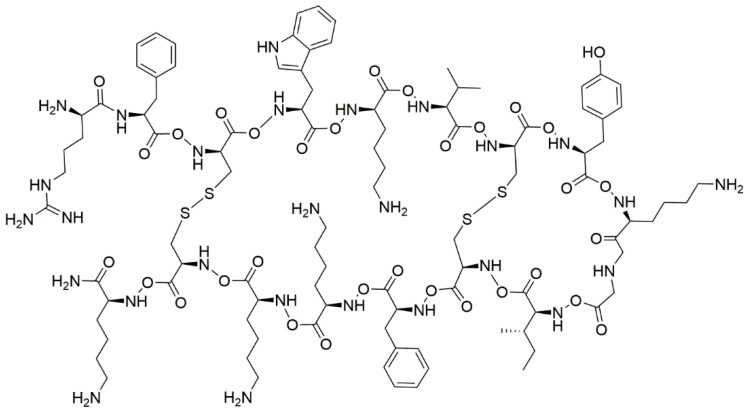
Structure of RP557 antimicrobial peptide.

For example, RP557 is an AMP, designed with the iterative chemical structure of the human cathelicidin LL-37, that showed a broad-spectrum antibacterial and antifungal activity (Figure 1). This AMP resulted to be active moderately against *Mab* with a MIC of 64 μg/mL [73]; most importantly, it was able to significantly impair the biofilm formation and increase the sensitivity of clarithromycin, cefoxitin, amikacin, and imipenem, even at lower concentrations (1/4 MIC). The evaluated dose is significantly lower than the minimal cytotoxic threshold of 128 μg/mL against mammalian cells, suggesting that RP557 could be a valid candidate to be integrated into the actual therapeutic regimen [73].

The effect against *Mab* growth of six AMPs that differ by only one amino acid each was also evaluated [74]. Two of them, AP1 (ILPWKWRWWKWRR) and AP3 (the D enantiomer of AP1), were active against *Mab* wt strain (ATCC 19977) and three clinical strains of *M. massiliense*, collected from CF individuals (MIC = 1.3–3.1 μg/mL). The other AMPs were not active. Since its promising result, AP1 was also evaluated against other 25 MABSC clinical strains, with both rough and smooth phenotypes, and from either CF or non-CF bronchiectasis patients, showing a similar MIC against all these strains, (1.5–6.2 μg/mL) [74]. Finally, different concentrations of AP1 (1–3 X MIC) were evaluated in a model of *Acanthamoeba castellani* infected with the three clinical isolates of *M. massiliense*. In this case, only the 3 × MIC concentration showed significant protection by amoebae against all the strains with no cytotoxic effect [74]. Despite good results, no cytotoxic tests were conducted to assess the safety of this AMP.

Non-Disulfide-Bridge-Peptide 5.5 (NDBP-5.5) is an amphipathic molecule derived from the *Hadrurus gertschi* scorpion venom, composed of 13 amino acids (IFSAIAGLLSNLL) [75]. It was proved to be quite active against *M. massiliense* in vitro, with a Minimal Bactericidal Concentration (MBC) of 266 μg/mL. Further investigations demonstrated its efficacy also in ex vivo and in vivo models. Firstly, the treatment with 200 μM NDBP-5.5 showed a 50% infection decrease on a macrophage infection model, similar to the treatment with clarithromycin 1.34 μM. Finally, this AMP lowered the *M. massiliense* load in the lungs and liver of infected IFN-γ KO mice when used at a dose of 2 mg/Kg, with a similar effect to clarithromycin 200 mg/Kg. Although NDBP-5.5 lowered the inflammatory reactions induced by the mycobacterial infection, unfortunately, it did not show a synergic effect in vitro with any evaluated drugs, nor did it completely clear the infection by itself [75].

Polydim-I (AVAGEKLWLLPHLLKMLLTPTP) is an AMP isolated from the poison of the Brazilian wasp *Polybia dimorpha*, which was proven to possess antimicrobial activity against *M. massiliense* with a MIC of 60.8 μg/mL [76]. The macrophage infection model showed a reduction of 40~50% mycobacterial load upon treatment with 7.6 μg/mL, far below the cytotoxic concentration observed in J774 cells of 121.5 μg/mL. Finally, Polydim-I was able to decrease mycobacterial burden by 90% in IFN-γKO infected mice at a dose of 2 mg/kg/mLW.

Polybia-II (WLKLGKMVIDAL) is another AMP isolated from the poison of the wasp *Pseudopolybia vespiceps* species that showed bactericidal and antifungal activity, and it was proven to be effective also against *M. massiliense* [77]. Indeed, Polybia-II was found to inhibit 80% of intracellular growth of *M. massiliense* in the macrophage infection model after 24 h of treatment with a dose of 12.5 μM, below the reported hemolytic effect on human red blood cells (EC_50_ = 48.47 μM) [77]. Despite promising results, the study lacked an in vivo evaluation in infected mice that could confirm the data.

All the studies regarding the use of AMPs against MABSC [74,75,76,77] suggest that these molecules may become an interesting addition to the therapeutic regimen to fight more efficiently MABSC infections. In fact, most of them not only showed antimycobacterial activity, mostly below the minimal cytotoxic dose but also are active either ex vivo or in vivo.

## 6. Innovation in Drug Delivery Systems

Organic and inorganic nanoparticles (gold, silver etc.), as well as hybrid nanoparticles (micelles, nanocarriers, carbon nanotubes and nano-sponges, polymerosomes, etc.), are all examples of nanomedicine tools, powerful drug delivery systems with the advantages of high efficiency and specificity and a sustained drug release over time at the target site. They have been successfully used to treat a plethora of diseases, from cancer to infections and as putative wound dressing [78,79,80].

Recently, those tools have been also investigated as putative weapons against *Mab*.

One example is gallium-based nanoparticles, which showed significant results in blocking *Mab* growth in a macrophage infection model, as well as in infected mice. Gallium shares some chemical–physical properties with iron, an important cofactor for many bacterial enzymes, essential for mycobacterial infection and survival inside their replicative niche, the macrophages vacuoles. Thus, blocking iron pathways may be a winning strategy to defeat mycobacterial infections [81,82,83,84]. Gallium, mimicking iron, is able to bind different iron-dependent proteins and to chelate molecules, such as the heme, with a similar affinity to iron itself [83]. Due to its properties, it can also bind bacterial proteins—for example, the siderophores—and for this reason, can be used to block the mycobacterial iron uptake pathway [84].

For example, the Gallium (III) *meso*-tetraphenylporphyrine chloride (GaTP) is active against *Mab* growth (MIC = 4–8 μg/mL for the ATCC 19977 reference strain). Unfortunately, GaTP is not able to penetrate macrophages, resulting in ineffective ex vivo [83] outcomes. Nevertheless, nanoparticles created by incapsulating GaTP with F127 polymer resulted to be significantly effective also in THP-1 macrophages infected with *Mab* at a concentration of 300 μM. Furthermore, when those nanoparticles were conjugated with mannose or folate, which bind specific macrophage receptors helping their internalization, the growth inhibition was significantly increased [83]. So far, the in vivo evaluation of infected mice to confirm the efficacy of gallium nanoparticles has not been conducted. Another study evaluated the effect of cationic chitosan-coated clarithromycin nanocapsules (CS-CLARI-NC) on both smooth and rough *Mab* morphotypes. Those nanocarriers, mainly composed of PLGA [poly (lactic-co-glycolic acid)], were able to significantly inhibit mycobacterial growth compared to the free drug on a murine macrophage ex vivo model, particularly with rough morphotype [85].

The efficacy of liposome encapsulated rifampicin compared to free rifampicin, in an ex vivo *Mab* infection model on differentiated human pro-monocytic THP-1 leukemia cells was evaluated. These liposomes, ~120 nm in size, showed time-dependent drug release, with most of the rifampicin released in the first 8 h, and full release in 24 h [86]. Testing was done on 3 different rifampicin concentrations, 24, 48 and 96 μM, resulting in a concentration-dependent inhibition of *Mab* growth that was significantly higher than the same concentration of the free drug [86].

Recently, Poerio et al. [87] showed that apoptotic body-like liposomes loaded with phosphatidylinositol 5-phosphate (ABL/PI5P) enhanced the anti-*Mab* response. This treatment in mice, intratracheally infected with *Mab*, resulted in about a 2-log reduction of pulmonary mycobacterial burden. Furthermore, the combination treatment with ABL/PI5P and amikacin resulted in a further significant reduction of both pulmonary mycobacterial burden and inflammatory response in comparison with the single treatments. This promising finding opens the possibility to use this regimen as a combined host- and pathogen-directed therapeutic strategy for the control of *Mab* infection.

Nanoniosomes are vesicles with a composition similar to that of the cell membrane (named niosomes) that have been loaded with antibiotics or lignin-silver nanoparticles. Nanoniosomes were not effective in vitro against *Mab* but could exhibit antibacterial activity in *Mab*-infected macrophages. For example, the nanoniosomes used to encapsulate ciprofloxacin or clarithromycin (ratio antibiotic-to-cholesterol 1:25) did not show ex vivo activity. On the contrary, rifabutin encapsulates nanoniosomes at 40 µg/mL exhibited an intracellular antibacterial effect that resulted in a 1.1 log reduction over 3 days using only one dose [88].

In conclusion, even if nanomedicine is a powerful tool used to successfully treat many diseases, only a few studies have been established to treat *Mab* infections via nanoparticles and nanocarriers. The combined use of ABL/PI5P liposomes and amikacin seems very promising [87], representing a successful example of a combined host- and pathogen-directed therapeutic strategy. It would be interesting if this path leads to new opportunities in the future since some promising results have already been achieved. 

## 7. Phytochemicals against Mycobacteria: A New Match May Begin

Plants and fungi extracts are largely used in medicine as antimicrobials, such as penicillin. Following this flow, different ongoing studies are evaluating new synthetic molecules but also poorly-studied phytochemicals, often looking for new sources like marine plants.

Some *Colletia paradoxa* extracts gathered in Brazil, for example, showed a weak inhibitory effect against *Mab* growth. The *C. paradoxa* extract RB 501has indeed a MIC of 312.5 μg/mL, while others from the same plant, like RB 508, showed a higher MIC of 1250 μg/mL. Similar activity has been reported for the same extracts also against *Mycobacterium fortuitum,* while lower MICs have been found against *M. massiliense* (78.12 μg/mL for RB 515, or 625 μg/mL for RB 507) [89]. The authors explained these results by hypothesizing that the lipophilic features of the analyzed flavonoids help them cross the mycobacterial membrane, making them effective against these microorganisms. Furthermore, they suggested that the mycobacterial fatty acid synthase II (FAS-II) enzyme cannot modify these extracts, owing to the presence of oxygen in the cyclic structure of the flavonoids [89].

A study conducted by Jiménez-Arellanes et al. in 2013 reported an MIC of 25 μg/mL against *Mab* for the chloroform extracts of the *Persea americana* seeds. The same extracts showed an MIC in the range of 25–100 μg/mL against other mycobacteria species, except for a rifampicin-resistant *Mtb* strain [90]. The ethanolic extracts from the same seeds also showed some antimycobacterial effect, but with higher MIC [90]. However, this evaluation lacks the single component analysis of the extracts that are useful in identifying the active metabolite against mycobacteria.

**Figure 2:**
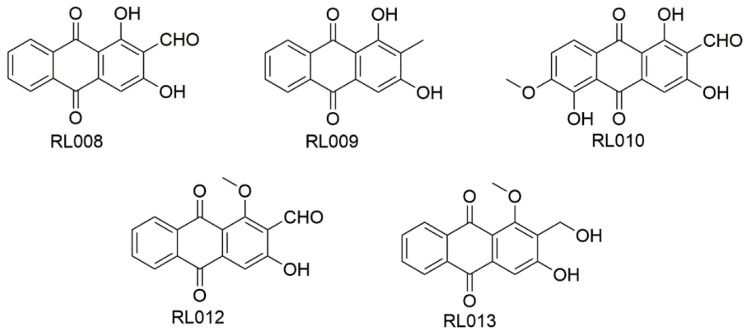
Chemical structure of the five fitochemicals isolated from *P. filamentosa*, showing activity against *Mab* strains.

More specific research analyses 22 pure compounds extracted from different parts of 4 medicinal plants (*Atalantia monophylla, Prismatomeris filamentosa, Ageratum conyzoides* and *Rhotmannia wittii*), and one extracted from the cultured mycelium of a luminescent mushroom (*Neonothopanus nambi*), against 30 MABSC clinical isolates (13 *Mab* and 17 *M*. *massiliense*). Five of the analyzed compounds [RL008 (nordamnacanthal), RL009 (rubiadin-1-methyl ether), RL011 (knoxiadin), RL012 (damnacanthal) and RL013 (damnacanthol)] (Figure 2), which derived from the roots of *P. filamentosa,* showed an MIC ranging from 1 to 128 μg/mL [89]. None of them showed any hemolytic effect on human red blood cells at the evaluated concentrations, and only low cytotoxicity on white cells [89]. Finally, two of them, RL008 and RL009, showed a synergistic effect with clarithromycin, reducing the MIC of this antibiotic up to 16 folds [91].

All the reported studies indicate that plants and fungi are still nowadays a source with a huge potential for discovering new molecules that are effective in fighting *Mab*.

## 8. Conclusions

In conclusion, this review provides an overview of the latest findings on innovative therapies against *Mab*. These strategies are not always designed to completely replace antibiotics, but to be a valuable aid to them, especially in enhancing their efficacy and shortening treatment time.

For some of the strategies described, positive results have been obtained at the early pre-clinical stages, and it is hoped that these will form the basis for developing these strategies and confirming their efficacy in vivo so that they can reach the clinical trial stage. In short, there is still a long way to go before these therapies can become part of the clinical treatment of these infections. At the same time, the development of new *Mab*-specific antibiotics is mandatory. Overall, a major commitment from the many stakeholders, as well as further financial efforts, are needed to develop an effective and curative therapy against *Mab* infections.

## Data Availability

Not applicable.
